# The Common Basis of Memory and Consciousness: Understanding the Brain as a Write–Read Head Interacting With an Omnipresent Background Field

**DOI:** 10.3389/fpsyg.2019.02968

**Published:** 2020-01-10

**Authors:** Joachim Keppler

**Affiliations:** Department of Consciousness Research, DIWISS, Roth, Germany

**Keywords:** declarative memory, memory formation, memory retrieval, conscious perception, self-referential consciousness, stochastic electrodynamics, zero-point field, superradiant phase transitions

## Abstract

The main goal of this article consists in addressing two fundamental issues of consciousness research and cognitive science, namely, the question of why declarative memory functions are inextricably linked with phenomenal awareness and the question of the physical basis of memory traces. The presented approach proposes that high-level cognitive processes involving consciousness employ a universal mechanism by means of which they access and modulate an omnipresent background field that is identified with the zero-point field (ZPF) specified by stochastic electrodynamics (SED), a branch of physics that deals with the universal principles underlying quantum systems. In addition to its known physical properties and memory capacities, the ZPF is hypothesized to be an immanently sentient medium. It is propounded that linking up to a particular field mode of the ZPF activates a particular phenomenal nuance, implying that the phase-locked coupling of a set of field modes, i.e., the formation of a so-called ZPF information state, constitutes an appropriate mechanism for the amalgamation of elementary shades of consciousness into a complex state of consciousness. Since quantum systems rest exactly on this mechanism, conscious memory processes in the brain are expected to differ from unconscious processes by the presence of the typical features of many-body quantum systems, particularly long-range coherence and attractor formation, which is supported by a huge body of empirical evidence. On this basis, the conceptual framework set out in this article paves the way for a new understanding of the brain as a write–read head interacting with the ZPF, leading to self-consistent interpretations of the neural correlates of memory formation and memory retrieval and explaining why these memory processes are closely intertwined with phenomenal awareness. In particular, the neural correlates suggest that the brain produces consciously perceived memory traces by writing sequences of information states into the ZPF and retrieves consciously experienced memory traces by reading sequences of information states from the ZPF. Using these theoretical foundations, altered states of consciousness and memory disorders can be traced back to impairments of the ZPF write–read mechanism. The mechanism should reveal itself through characteristic photon emissions, resulting in testable predictions.

## Introduction

Our memory endows us with absolutely amazing faculties. We are permanently exposed to a stream of external stimuli that we process and consciously perceive. Immediately after going through a chain of events, we are able to recall the sequence of our experiences, all charged with a rich spectrum of phenomenal qualities. And even months or years later, we can often vividly remember all the details.

Each of us is familiar with this ability that falls into the category of autobiographical memory, a subtype of episodic memory. In conjunction with our semantic memory, which, for example, relates to the storage of words, the episodic memory forms a particular class of high-level cognitive functions, termed declarative memory. A plethora of empirical findings indicates that declarative memory is associated with consciousness, or more specifically, that *both the formation and retrieval of episodic and semantic memories are inextricably linked with phenomenal awareness* (Tulving, 1985; Behrendt, 2013), suggesting that the neural correlates of memory formation (NCMf) are congruent with the neural correlates of conscious perception (NCCp), while it can be expected that the neural correlates of memory retrieval (NCMr) are in accordance with the neural correlates of self-referential consciousness (NCCsr). However, even though, as we will see, these one-to-one correspondences are well supported by a huge body of neuroscientific evidence, the neural mechanisms involved give no concrete indication and satisfying explanation as to why declarative memory functions are always accompanied by phenomenal qualities (qualia). Thus, a serious explanatory gap remains (Levine, 1983; Chalmers, 1995, 1996), due to the fact that the phenomenal properties of a system cannot be simply reduced to structural, functional, or organizational principles, no matter what type of structure, function, or organization is involved. This leads to the challenge of integrating qualia into the modern scientific worldview and explaining the occurrence of phenomenal properties in high-level cognitive functions.

Closely related to the mechanisms underlying memory and consciousness is the question of the locus and the substrate of memory. In this context, the concept of the *memory trace* or *engram* is commonly used to denote the physical representation of memories (Lashley, 1950; Hübener and Bonhoeffer, 2010; Eichenbaum, 2016; Poo et al., 2016). According to common doctrine, the engram is associated with structural changes in the involved brain regions, or more precisely, the persistent modification of neuronal connections via long-term potentiation (LTP) and long-term depression (LTD) is deemed the primary mechanism of memory formation (Hübener and Bonhoeffer, 2010; Poo et al., 2016). However, research on artificial neural networks suggests that this widespread view is afflicted with serious inconsistencies. After all, memory formation must not be confused with learning where the rewiring of neural networks serves as a mechanism to adapt to new or changing sensory inputs. In such learning tasks, the network occupies the function of a complex adaptive pattern recognition system with the purpose of mapping incoming signals on neural activity patterns in such a way that similar input signals are represented by similar neural activity patterns (Kohonen, 1982). A central aspect of the learning process is to present sequences of different input signals to the network. After completion of the training process, the neural network allows for a fine-grained discrimination of input signals. It cannot be denied that neural networks are highly efficient and powerful in the execution of such classification tasks in which patterns need to be separated or completed in order to distinguish between individual inputs. But the crucial point is that the sequence of input signals (events) the network has seen during the learning process is not stored anywhere in the network, meaning that the neural network itself has no episodic memory. Translated to the brain, it is hardly conceivable that one and the same network architecture can act as both a high-performance discriminator of sensory inputs and a long-term storage system for episodic memories. This casts doubt on the neural foundation of memory and raises the question of the true physical basis of engrams.

The main goal of this work consists in addressing the two fundamental issues of consciousness research and cognitive science pointed out above. First, there is the question of why declarative memory functions are closely intertwined with phenomenal awareness. The challenge here is to develop a conceptual framework that is capable of disclosing the common mechanisms underlying memory and conscious processes, thus unveiling the deeper connection between the recording of events and the attachment of phenomenal qualities to these events. Second, it has to be clarified which medium the storage of memory traces is based on, resulting in the requirement to specify the physical basis of engrams.

In order to tackle these two core questions, a novel approach is being pursued that builds on previous works (Keppler, 2012, 2013, 2016, 2018a,b; Shani and Keppler, 2018). It accepts consciousness as ontologically fundamental, i.e., an irreducible feature of ultimate reality, and postulates that the entire phenomenal color palette is grounded in a ubiquitous, modifiable background field functioning as a cosmic storage medium (substrate). Following this line of thought, it is hypothesized that high-level cognitive processes involving consciousness employ a universal mechanism by means of which they access and modulate this omnipresent, immanently sentient substrate. As it will turn out, the edifice of modern physics can offer not only a promising candidate for the background field, but also an appropriate modulation mechanism. On this basis, the empirical body of evidence can be interpreted such that in the stimulus-oriented operating mode the brain produces consciously perceived memory traces by imprinting sequences of information states on the substrate. In the stimulus-independent operating mode, the brain is receptive to previously generated information states constituting the record of conscious experiences, suggesting that memory traces are retrieved by reading sequences of information states from the substrate. It is concluded that the presented approach paves the way for a new understanding of the brain as a write–read head interacting with an all-pervading background field, resulting in self-consistent interpretations of the neural correlates of memory and consciousness and explaining why declarative memory processes are always linked to phenomenal awareness. Beyond its explanatory power, the approach gives fresh impetus to the field of cognitive science by being able to define a new research program that aims at a direct confirmation of the fundamental mechanisms underlying memory and consciousness.

The article is organized in such a way that the next section outlines the conceptual framework for consciousness and memory. This framework forms the theoretical foundation for all ensuing considerations. Thereupon, we get into the interpretation of the empirical findings, first looking at the NCMf and NCCp, then turning to the NCMr and NCCsr. In the subsequent discussion, we draw some additional conclusions arising from the presented approach, address working memory and altered states of consciousness, and touch on further questions that go beyond the scope of the present paper. The final section is dedicated to future perspectives and testable predictions.

## Conceptual Framework for Consciousness and Memory

In order to embed consciousness in a self-consistent conceptual framework that is compatible with all laws of physics, it seems natural and reasonable to resort to quantum field theory (QFT). The relevance of QFT to consciousness research is based in a wider sense on the fact that field theories as such form the bedrock of our current understanding of the universe, and in a narrower sense on the fact that QFT is the key to a deeper understanding of the dynamics of biological systems. The latter statement runs counter to the widespread belief that classical physics should be sufficient to explain the dynamical properties of such systems. However, the proponents of classical approaches completely ignore that the long-range order phenomena and dissipative structures characteristic of living matter in general and neural activity patterns in particular cannot be accounted for without the theoretical background of QFT (Del Giudice et al., 1985; Freeman and Vitiello, 2006; Lloyd, 2011). At this point, it is important to emphasize that QFT goes far beyond ordinary quantum mechanics. While quantum mechanics is limited to a special class of systems, basically stationary systems with a fixed number of particles, QFT offers the full formalism for the description of all types of complex systems, most notably systems in which the charged components interact with the electromagnetic field. It is precisely this matter–field interaction that causes the collective behavior of the system components and is responsible for the formation of *macroscopic quantum systems* which are not to be understood as simple accumulations of microscopic quantum systems (atoms and molecules), but rather as integrated wholes whose macroscopic properties are governed by the laws of quantum physics, as manifested in the guise of long-range coherence, pattern formation, and phase transitions. As we will see, these macroscopic organizational phenomena play a crucial part in the mechanisms underlying memory and conscious processes, giving rise to the important role of QFT in consciousness research.

The approach followed here is based on a branch of research that deals primarily with the foundations of QFT. In short, the ultimate ambition of this branch, known as stochastic electrodynamics (SED), is to understand the occurrence of quantum phenomena and the behavior of quantum systems on the basis of universal principles, thereby revealing the deeper level of reality behind the formalism of QFT (Marshall, 1963, 1965; Boyer, 1969, 1975; De la Peña-Auerbach and Cetto, 1977; De la Peña and Cetto, 1994, 1995, 1996, 2001, 2006; Cetto et al., 2012; De la Peña et al., 2009, 2015). Thus, SED provides a unique view of the fundamental mechanisms underlying our world.

**FIGURE 1:**
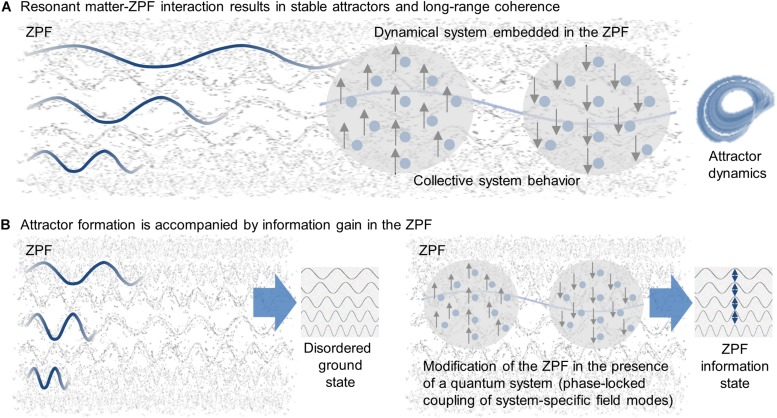
Universal principles underlying quantum systems. **(A)** Every material system is embedded in the zero-point field (ZPF) that constitutes an omnipresent ocean of energy. Due to their continuous coupling with ZPF, the electrically charged building blocks of a system behave like driven oscillators. Sufficient shielding against disturbing effects provided, a system enters the quantum regime and falls into a stable attractor, each system being characterized by a specific set of resonance frequencies filtered out of the full ZPF spectrum. For many-body systems, the resonant interaction with the ZPF results in long-range coherence and collective system behavior. **(B)** While in its ground state the ZPF is a completely disordered field with uncorrelated field modes, the resonant interaction with a quantum system has repercussions on its internal structure. The formation of an attractor is accompanied by the phase-locked coupling of the set of field modes that correspond to the system’s resonance frequencies, resulting in an ZPF information state that has higher information content than the disordered ground state of the ZPF.

An essential cornerstone of SED is the *zero-point field* (ZPF) that is construed as a real, all-pervading, electromagnetic background field meeting all basic symmetry requirements, such as homogeneity, isotropy, and scale invariance (De la Peña and Cetto, 1994, 1995). Illustratively, the ZPF can be thought of as a vibrant, ubiquitous ocean of energy whose initial state is completely disordered, meaning that the individual modes of the field, i.e., the individual components of the frequency spectrum, have no correlation with each other. In this form, the ZPF functions as an omnipresent substrate permanently interacting with the electrically charged constituents of any material system. Owing to their continuous coupling with the energy field, the constituents behave like driven stochastic oscillators, each system being characterized by a specific set of resonance frequencies filtered out of the full ZPF spectrum (De la Peña et al., 2015). If a system is sufficiently shielded against disturbing effects and the dynamics of the system are governed by the interaction of its constituents with the ZPF, an attractor state is reached and a stable energetic equilibrium is established in which the average power radiated by the system is exactly compensated by the average power the field feeds into the system. It turns out that these attractor states correspond to the stationary states described by quantum physics (De la Peña and Cetto, 1995, 2001, 2006). In other words, the peculiar behavior of a quantum system is due to its interaction with the background field, which imposes dynamic boundary conditions on the system that are reflected in quantization rules. In this way, *the ZPF acts as an orchestrator behind the scenes, being instrumental in the formation and stabilization of attractors*. For many-body systems, this orchestration results in *long-range coherence* and collective system behavior (De la Peña and Cetto, 2001), as depicted in Figure 1A.

It is now of crucial importance that the dynamic interaction between a quantum system and the background field has repercussions on the internal structure of the field. More precisely, the achievement of a dynamic equilibrium state is connected with the phase-locked coupling of the set of field modes that correspond to the system’s resonance frequencies (De la Peña and Cetto, 2006; De la Peña et al., 2009). Consequently, the formation of an attractor is always associated with an increase in order in the ZPF, i.e., each attractor is accompanied by a partially ordered ZPF state that has higher information content than the disordered ground state of the ZPF. Therefore, in the following, such an attractor-specific ZPF state is referred to as *ZPF information state* (Keppler, 2012, 2016). As far as many-particle systems are concerned, it can be summarized that due to the dynamic matter–field interaction, *each long-range coherent state is associated with a specific ZPF information state* (see Figure 1B).

Moreover, a closer look at the equation of motion for a system embedded in the background field unveils its non-Markovian character, meaning that the stochastic processes underlying quantum systems are processes with long-term memory (De la Peña-Auerbach and Cetto, 1977). This finding is corroborated by experimental and theoretical investigations of pilot-wave systems, showing that path memory is decisive for the emergence of quantum phenomena (Fort et al., 2010; Bush, 2015). These insights emphasize the importance of memory in explaining the dynamics of quantum systems and support the conclusion that *ZPF information states arising from attractor formations are persistent states*.

The aforementioned unique properties of the ZPF give well-founded reason to assume that this ubiquitous background field, in addition to its functions as inexhaustible energy reservoir and substrate of memory, could also play the role of a cosmic field of consciousness (Keppler, 2012, 2016; Shani and Keppler, 2018). This conjecture is based on the idea that the ZPF is an immanently sentient medium, or more specifically, a dual-aspect entity with an outer physical manifestation and an inner phenomenal essence. The ZPF in its undisturbed ground state can thus be understood as a formless, undifferentiated sea of consciousness all conceivable phenomenal shades are inherent in. So, what manifests itself externally as a field mode with definite frequency and energy is presumably charged with a particular conscious experience, entailing that the selection of a specific field mode activates a specific phenomenal nuance. Therefore, the universal mechanism behind quantum systems described above is perfectly suited for tapping into the cosmic field of consciousness and merging selectively filtered elementary shades of consciousness into complex states of consciousness, leading to the hypothesis that *each ZPF information state, i.e., each set of phase-locked ZPF modes, is associated with a conscious state that is preserved in the field* (Keppler, 2012, 2013, 2016, 2018b). As a consequence, an essential requirement to be met by a conscious system is that it must be able to form transiently stable coherent states (attractors), each of which is accompanied by an attractor-specific ZPF information state. According to this reasoning, all conscious systems are necessarily quantum systems, since classical systems are not coupled with the ZPF and therefore cannot influence the internal structure of the ZPF. In other words, the presented approach suggests that *the basic principle behind conscious systems equipped with memory consists in the modification of an omnipresent background field of consciousness*. With that said, conscious memory processes in the brain should differ from unconscious processes by the presence of the typical characteristics of many-body quantum systems, most notably the formation of transiently stable attractors exhibiting long-range coherence (Keppler, 2012, 2013, 2016, 2018b).

The advantage of this view lies in the seamless integration of consciousness into the causal network of relationships underlying the dynamics of quantum systems and thus in the elimination of the explanatory gap. In conventional materialist approaches, the gap arises because certain neural activity patterns are held responsible for the generation of consciousness *per se*, which entails an ontological discontinuity. For the emergence of a systemic consciousness from insentient system components undoubtedly implies a mysterious creation mechanism that is completely non-transparent in its mode of action. In contrast, in the scenario presented here, the organizational principles underlying conscious processes are not accountable for the generation of consciousness *per se*, but for the modulation and selective restriction of a cosmic field of consciousness (Shani and Keppler, 2018). The proposed modulation mechanism is intelligible and completely transparent.

## Neural Correlates of Memory Formation and Conscious Perception

Building on the conceptual framework set out in the previous section, we turn to the discussion of the NCMf and NCCp.

Starting with the NCMf, the empirical findings suggest that successful memory encoding relies on significant increases in theta and gamma power, being indicative of theta and gamma phase synchrony (Klimesch et al., 1996; Axmacher et al., 2006). This applies to the formation of semantic and episodic memories, both of which are correlated with enhanced large-scale synchronization of cortical network activity in the theta and gamma frequency band (Weiss and Rappelsberger, 2000; Sederberg et al., 2003), exhibiting also theta-phase to gamma-amplitude coupling (Friese et al., 2013). In addition, it turns out that memory encoding is based on the dynamic interplay between the neocortex and the hippocampus. More precisely, declarative memory formation is related to considerable increases in neocortical and hippocampal gamma power (Sederberg et al., 2007) and to cortico-hippocampal theta oscillations (Lega et al., 2012), with the episodic and semantic memory performance being determined by the degree of theta synchronization (Klimesch, 1999). Taken as a whole, the experimental findings imply that an episodic memory trace is partitioned into a sequence of events. The encoding of each individual event relies on transient gamma phase synchronization of hippocampal and cortical regions, while the periodic integration and disintegration of the synchronized activity patterns is reflected in the theta rhythm (Nyhus and Curran, 2010), indicating that the synchronized interaction between cortical areas and the hippocampus fuses various sensory-specific aspects of an event into a coherent memory representation (Werkle-Bergner et al., 2006).

Proceeding to stimulus-induced conscious perception and, hence, to the key characteristics of the NCCp, the empirical data reveal that our streams of phenomenal awareness are based on the recurring formation and dissolution of highly synchronized large-scale activity patterns in the cortex. The phase synchronization of the cell assemblies participating in the activity patterns lies in the gamma frequency band, while the repetition rate of these transiently stable patterns follows the theta cycle (Desmedt and Tomberg, 1994; Rodriguez et al., 1999; Engel and Singer, 2001; Melloni et al., 2007; Doesburg et al., 2009; Gaillard et al., 2009). Taken together, the experimental insights suggest that a stream of conscious perception is represented by a sequence of conscious moments, with each individual conscious moment being related to transient gamma phase synchronization of widely-separated cortical regions and the periodic integration and disintegration of the synchronized activity patterns being reflected in the theta rhythm (Doesburg et al., 2009; Singer, 2015).

In summary, the body of evidence corroborates the high degree of congruence between the NCMf and NCCp and gives rise to the conclusion that both the formation of declarative memories and the conscious awareness of sensory stimuli rest on recurrent gamma synchronization of distributed neuronal assemblies (Axmacher et al., 2006; Jensen et al., 2007). Taking into account the role of the theta rhythm and the importance of the hippocampus in both processes, it seems natural to infer that conscious perception is episodic memory formation “in action” (Behrendt, 2013).

A deeper analysis of the data reveals that the observed patterns of long-range coherent network dynamics represent attractors in an attractor landscape, with the striking characteristic that immense numbers of neurons shift simultaneously and abruptly between consecutive attractors (Freeman, 1991, 2005, 2007). The existence of such scale-free neuronal avalanches clearly indicates that the processes of macroscopic pattern formation are due to critical phenomena, i.e., second-order phase transitions, occurring at theta rates (Freeman et al., 2003; Freeman, 2004, 2005, 2007; Freeman and Vitiello, 2006, 2007; Cocchi et al., 2017). Each theta cycle takes place in such a way that an appropriate sensory stimulus triggers a reorganization of the background activity, culminating almost instantaneously in a coherent activity pattern. This pattern dissolves after a few hundred milliseconds and is superseded by a short period of disorder that in turn creates the preconditions for the next phase transition and, thus, the formation of the subsequent attractor (Freeman, 2009). It is indisputable that the proper description of such phase transitions and critical phenomena requires the formalism of QFT (Zinn-Justin, 1996; Freeman and Vitiello, 2006, 2007).

**FIGURE 2:**
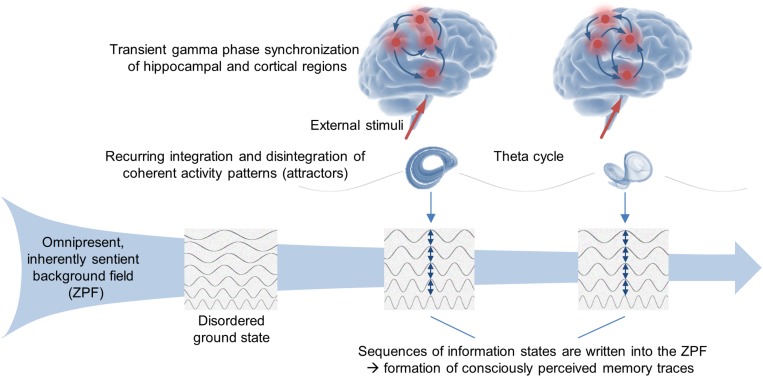
Stimulus-oriented operating mode of the brain. A huge body of empirical evidence suggests that declarative memory formation and conscious perception are based on one and the same fundamental mechanism. Concretely, an episodic memory trace is partitioned into a sequence of events. The encoding of each individual event relies on transient gamma phase synchronization of hippocampal and cortical regions, while the periodic integration and disintegration of the synchronized activity patterns (attractors) is reflected in the theta rhythm. From the perspective of the SED-based conceptual framework, each attractor formation is accompanied by a persistent, attractor-specific ZPF information state that is associated with a specific conscious state. Accordingly, the experimental findings support the hypothesis that in the stimulus-oriented operating mode the brain produces consciously perceived memory traces by writing sequences of information states into the ZPF.

Harking back to the SED-based conceptual framework outlined above, which provides insight into the deeper reality behind the formalism of QFT and incorporates consciousness in the form of an omnipresent, inherently sentient background field, we arrive at a self-consistent interpretation of the NCMf and NCCp, implying that the ZPF is crucially involved in the formation of transiently stable attractors, that each attractor is accompanied by a persistent, attractor-specific ZPF information state, and that each ZPF information state is associated with a specific conscious state. Accordingly, the NCMf and NCCp suggest that *in the stimulus-oriented operating mode the brain produces consciously perceived memory traces by writing sequences of information states into the ZPF*, which is illustrated in Figure 2.

## Neural Correlates of Memory Retrieval and Self-Referential Consciousness

After having discussed the deeper connection between memory formation and conscious perception, we perform an analogous analysis of the stimulus-independent cognitive processes relating to consciousness, reflected in the NCMr and the NCCsr.

Looking at the NCMr, the empirical findings are indicative of the involvement of resting-state networks, particularly the default mode network (DMN) that comprises several distributed cortical regions, such as the medial prefrontal cortex (MPFC), the posterior cingulate cortex (PCC), and the precuneus (Raichle et al., 2001; Greicius et al., 2003). It turns out that DMN activation is linked to autobiographical memory (Spreng and Grady, 2010; Peters et al., 2013). To be more precise, both the DMN and the hippocampus show increased activity during successful episodic memory retrieval (Huijbers et al., 2011), with the close coupling of parietal DMN and hippocampal regions becoming apparent from studies examining functional connectivity (Sestieri et al., 2011). Overall, there is strong evidence that the role of the hippocampus in episodic memory recall consists in multimodal binding, meaning that the hippocampus fuses the distinct aspects of an event into a holistic memory representation, or in more precise terms, that the re-experiencing of an events relies on the formation of an attractor involving the hippocampus (Horner et al., 2015). As far as the neurophysiological characteristics of the corresponding activity patterns are concerned, the data reveal that the retrieval of semantic long-term memories is associated with oscillations in the alpha frequency band (Klimesch, 1999) and that autobiographical remembering is related to substantial alpha power increases observed in the DMN, the hippocampus, and content-specific cortical regions (Knyazev et al., 2015).

Turning to stimulus-independent thought and self-referential conscious experience and, hence, to the core features of the NCCsr, the experimental findings point to significant activations of the DMN (Gusnard et al., 2001; Buckner and Carroll, 2007; Mason et al., 2007) and corroborate that particularly the PCC and MPFC in combination with the hippocampus are the key structures participating in self-referential mental processes (Behrendt, 2013). As has been shown, these processes are associated with enhanced alpha activity and long-range alpha synchrony as well as increased gamma power and gamma synchrony in the DMN regions (Mantini et al., 2007; Jann et al., 2009; Knyazev et al., 2011; Knyazev, 2013).

In summary, the body of empirical evidence substantiates the high degree of congruence between the NCMr and the NCCsr and supports the conclusion that self-referential conscious awareness is episodic memory retrieval “in action” (Behrendt, 2013). Consequently, the experimental insights suggest that the stream of self-referential consciousness, tantamount to the retrieval of declarative memories, is partitioned into sequences of events. The recall of each individual event is based on transient gamma phase synchronization of DMN and hippocampal regions, while the periodic integration and disintegration of the synchronously oscillating cell assemblies is reflected in the alpha rhythm. Put another way, the alpha cycle originates from the recurring formation and dissolution of transiently stable attractors, termed microstates, which undergo rapid transitions and exhibit scale-free dynamics (Lehmann et al., 1987, 1998; Britz et al., 2010; Van de Ville et al., 2010). A deeper analysis of these patterns of large-scale coherent network activity points to a diverging correlation length with increasing pattern size (Fraiman and Chialvo, 2012) and demonstrates that their dynamical properties are characteristic of systems close to the critical point of a second-order phase transition (Tagliazucchi et al., 2012; Cocchi et al., 2017).

**FIGURE 3:**
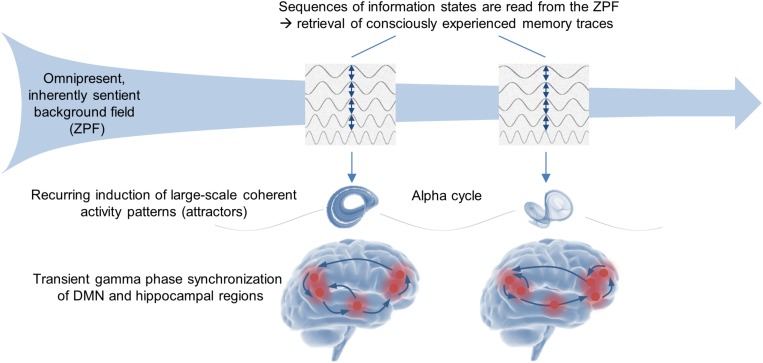
Stimulus-independent operating mode of the brain. The experimental insights suggest that the retrieval of declarative memories and the stream and self-referential consciousness rest on one and the same fundamental mechanism. In concrete terms, the recall of each individual event of a memory trace is based on transient gamma phase synchronization of DMN and hippocampal regions, while the recurring integration and disintegration of the synchronously oscillating cell assemblies is reflected in the alpha rhythm. Interpreted from the viewpoint of the SED-based conceptual framework, the brain is receptive to previously generated sequences of ZPF information states that constitute existing memory traces, with each ZPF information state inducing a phase transition that results in a large-scale coherent DMN-hippocampal activity pattern (attractor). Accordingly, the empirical body of evidence supports the hypothesis that in the stimulus-independent operating mode the brain retrieves consciously experienced memory traces by reading sequences of information states from the ZPF.

As with the processes of memory formation and stimulus-induced conscious perception, these results indicate that an in-depth understanding of memory retrieval and self-referential conscious processes requires the theoretical background of QFT and the explanatory framework of SED. Accordingly, the NCMr and NCCsr can be interpreted in such a way that in the self-referential operating mode the brain is receptive to previously generated sequences of ZPF information states that constitute existing memory traces. Each ZPF information state induces a phase transition that results in a large-scale coherent DMN-hippocampal activity pattern. Hence, the NCMr and NCCsr suggest that *in the stimulus-independent operating mode the brain retrieves consciously experienced memory traces by reading sequences of information states from the ZPF*, which is depicted in Figure 3.

## Discussion

In a nutshell, the presented approach proposes that high-level cognitive processes involving consciousness employ a universal mechanism by means of which they access and modulate an omnipresent substrate. This substrate is identified with the ZPF that, in addition to its known physical properties and memory capacities, is hypothesized to be the ground of all conceivable shades of phenomenal awareness. It is propounded that linking up to a particular mode of the ZPF activates a particular phenomenal nuance, implying that the phase-locked coupling of a set of field modes constitutes an appropriate functional principle for the amalgamation of elementary shades of consciousness into a complex, multi-faceted state of consciousness. Since quantum systems rest exactly on this functional principle, conscious memory processes in the brain are expected to differ from unconscious processes by the presence of the typical features of many-body quantum systems, particularly long-range coherence and attractor formation, which is supported by a huge body of empirical evidence.

On this basis, the conceptual framework set out in this article paves the way for a *new understanding of the brain as a write–read head interacting with the ZPF*, resulting in self-consistent interpretations of the NCMf and NCMr and explaining why these memory processes are inextricably linked with phenomenal awareness. More specifically, a fresh perspective emerges according to which the brain produces consciously perceived memory traces by writing sequences of information states into the ZPF and retrieves consciously experienced memory traces by reading sequences of information states from the ZPF. As far as the storage medium of memory traces is concerned, the picture takes shape that the physical basis of engrams is the ZPF and that *an engram consists in a sequence of ZPF information states*. At a very abstract level, this results in an analogy to a tape recorder in which the write head (by means of magnetic alignment) has the task of generating ordered states on a magnetic tape that can be retrieved in the read mode. In the case of conscious memory processes, the ZPF plays the role of the tape, while the generation of order is accomplished by the phase-locked coupling of sets of ZPF modes.

The existence of two clearly separated and mutually exclusive cerebral operating modes, namely, stimulus-induced perception on the one hand and stimulus-independent thought on the other hand, has been underpinned by numerous studies. The main clues pointing to these opposite functions consist in the anticorrelation between task-positive networks and the DMN (Gusnard et al., 2001; Fox et al., 2005; Fransson, 2005; Uddin et al., 2009), the negative correlation between frontal theta oscillations and DMN activity (Scheeringa et al., 2008), as well as the task-related suppression of gamma oscillations in the PCC and MPFC (Jerbi et al., 2010). Following the line of thinking presented here, these characteristics reflect the switching between the ZPF write and ZPF read mode of the brain (Keppler, 2018b).

Working memory processes combine both operating modes. For one thing, this is expressed in increased activity in the alpha band and the correlation of the alpha power with the number of items held in memory (Jensen et al., 2002). For another thing, the data reveal that, in addition to synchronized alpha activity, working memory maintenance manifests itself in significantly enhanced frontal theta oscillations, as indicated by systematic increases of the theta power with growing working memory loads (Khader et al., 2010). Thus, working memory tasks geared to the temporary maintenance of information for processing purposes comprise, as one would expect, conscious memory formation and memory retrieval processes. Employing the conceptual framework at hand, this can be interpreted in such a way that a typical working memory process includes both the encoding of information in the ZPF and the read-out of information from the ZPF. Some recent studies claim to provide evidence for unconscious working memory, indicating that under certain circumstances there may be a partial dissociation between working memory and conscious processes (Soto et al., 2011; Bergström and Eriksson, 2014, 2015, 2018; Dutta et al., 2014; Trübutschek et al., 2017). However, this evidence has been critically examined and questioned by other groups (Stein et al., 2016; Persuh et al., 2018). Given the present lack of consensus on the unambiguous definition of the working memory concept and the unavailability of a clear demarcation of the neural correlates of working memory from interfering high-level cognitive processes, this topic will not be further discussed in this work but, rather, postponed until the neurophysiological signatures pertaining to working memory are pinpointed.

Furthermore, the theoretical foundations laid in this work add up to a satisfying explanation of altered states of consciousness. According to the ideas set forth in the previous sections, such states are expected to occur if the fundamental mechanisms underlying ordinary stimulus-induced and self-referential conscious processes are impaired. This is, for instance, the case under hypnotic conditions where the establishment of large-scale coherent network dynamics and attractors is suppressed (Kallio and Revonsuo, 2003; De Pascalis et al., 2004; De Pascalis, 2007; Fingelkurts et al., 2007; Miltner and Weiss, 2007; Jamieson and Burgess, 2014), indicating that the ZPF write mechanism is disrupted. As a result, attractor-specific ZPF information states cannot be generated, implying that the conscious perception of external stimuli is prevented (Keppler, 2018a). On the other hand, significantly decreased levels of neural activity and synchrony in DMN regions through to broadband disintegrations of the DMN can be observed in long-term meditators (Brewer et al., 2011; Fingelkurts et al., 2016; Panda et al., 2016) as well as under the influence of psychedelics (Carhart-Harris et al., 2012, 2016; Muthukumaraswamy et al., 2013; Palhano-Fontes et al., 2015), indicating that under such conditions the ZPF read mechanism is seriously disturbed. As a consequence, previously generated sequences of ZPF information states cannot be accessed, large-scale coherent DMN-hippocampal activity patterns are not induced, and self-referential conscious states are not instantiated, leading to ego dissolution (Keppler, 2018b). Using the same line of argument, one can give reasons why damage to DMN regions is linked to substantial impairments in the retrieval of autobiographical memories, as corroborated empirically (Philippi et al., 2015). It should be emphasized that this explanatory approach distinguishes itself from other attempted explanations in being capable of covering all insights about altered states of consciousness. For example, the spiritual experiences provoked by psychedelics, such as “feelings of profound joy and peace” and a “sense of oneness with the world” (Carhart-Harris et al., 2014), cannot be plausibly construed as products of brain activity, considering the finding that “no increases in oscillatory power were observed in any region” (Muthukumaraswamy et al., 2013). Rather, these observations suggest that under normal conditions, the cerebral read head “is attuned, and hence restricted, to a limited spectrum of ZPF modes, while meditative practices and psychedelics remove these restrictions” (Keppler, 2018b). From this perspective, spiritual experiences reflect the awareness of the unrestricted ground state of the ZPF.

At this point, a note is appropriate with regard to the classification of the write and read mechanisms into the categories *normal* and *disrupted*. According to the reading given above, *normality* refers to the proper functioning of the brain under consideration of existing environmental factors and, thus, to survival in everyday life, which can only be reasonably ensured if the write and read mechanisms are geared to each other. Solely under these conditions, i.e., if the read mechanism is adjusted to the spectrum of ZPF modes used for the encoding of memory traces, sequences of information states written into the ZPF can be read out later, allowing the retrieval of memories. From this point of view, altered states of consciousness, particularly ego dissolution and spiritual experiences, which arise under the application of meditation techniques or under the influence of psychedelics are to be understood as deviations from the norm and thus as *disruptions* of the normal state. Interestingly, however, meditative practices as well as psychedelics and the associated altered states of consciousness can trigger transformation processes in the DMN regions that lead to sustained modifications of the functional connectivity and to lasting positive behavioral changes, resulting in beneficial therapeutic effects (Smigielski et al., 2019). On the basis of the theoretical framework presented here, these findings can be interpreted in such a way that meditative and psychedelic experiences have repercussions on the structure of the cerebral write–read head (see also next paragraph), which may cause a rewiring of formerly dysfunctional brain areas and facilitate the recovery of the properly functioning ZPF read mechanism.

In the final part of the discussion, we revisit the relationship between synaptic plasticity and memory. As already mentioned earlier, the view is widely held that the storage of information is associated with structural alterations in the involved brain regions (Hübener and Bonhoeffer, 2010), that molecular modifications at all levels of the brain structure play a critical role in long-term memory formation (Bisaz et al., 2014), and particularly that engrams are represented by LTP- and LTD-caused persistent changes in the strength of synaptic connections (Poo et al., 2016). However, this prevailing doctrine is countered by skeptical views, according to which there is so far no compelling evidence substantiating the direct link between LTP and memory and that “it remains to be clearly shown that induction of LTP will result in some form of memory consolidation” (Lynch, 2004). The conceptual framework set out in this article shares this skepticism by pointing to a fundamental distinction between memory encoding and learning, as suggested by the study of artificial neural networks that function as complex adaptive pattern recognition systems. In networks of this sort, the learning process consists in a continuous rewiring in such a way that incoming signals are topologically mapped on neural activity patterns, allowing the network to incorporate new sensory inputs and adapt to changes in the environment (Kohonen, 1982). This continuous updating process ensures sustained high efficiency and selectivity in classification tasks and, hence, fine-grained discrimination of input signals. But by no means does such a network have an episodic memory, meaning that the sequence of events the network has seen during the updating process is not stored anywhere in the network structure. Rather, the retention of episodic memory traces requires a separate storage medium the neural network communicates with. In the present scenario, as far as the physical brain is concerned, the ZPF plays the role of this medium. Regarding further developments, a full-blown theory of memory formation and retrieval, which is beyond the scope of this article, must include the repercussion of new experiences on the brain structure. In this context, the understanding of the different sleep phases is of major importance, especially as experimental findings indicate that long sequences of newly acquired highly synchronous activity patterns are replayed during slow-wave and REM sleep and may result in synaptic plasticity in the hippocampus and neocortex (Axmacher et al., 2006). For the time being, the theoretical foundations discussed above support the hypothesis that ongoing structural modifications reflect the adaptation of the ZPF write–read head to the ever-changing stream of sensory stimuli.

## Future Perspectives

In the concluding section, we want to take a closer look at the fundamental mechanism underlying conscious memory processes. As we have seen, there is ample evidence that second-order phase transitions play a significant role in these processes, which manifests itself in the occurrence of scale-free neuronal avalanches where immense numbers of neurons shift simultaneously and abruptly between attractors. The transition between two consecutive attractors, which are ordered states exhibiting long-range coherence, is indicated by a short period of disorder. According to the conceptual framework set forth in this work, the whole transition process goes along with a reorganization of the ZPF in such a way that the formation of an attractor leaves a characteristic fingerprint, i.e., an attractor-specific information state, in this omnipresent background field. In order to confirm the proposed mechanism behind conscious processes, experimental proof has to be provided that the ZPF plays a decisive role in the periodic formation of coherent activity patterns and that it is modified as a result of its dynamic interaction with neural networks. The planning and preparation of suitable experiments requires an understanding of the recurring phase transitions the mechanism is based on. In the following, a first impression of the theoretical background and a first indication of the resulting experimental strategy will be given.

In concrete terms, it is reasonable to expect that the transition from a disordered to an ordered state, characterized by the presence of a coherently oscillating cell assembly, is a so-called *superradiant phase transition* that occurs in the case of sufficiently strong coupling between a many-body system and the electromagnetic background field and is accompanied by a photon pulse originating from the collective emission of radiation (Dicke, 1954; Hepp and Lieb, 1973; Wang and Hioe, 1973). More precisely, when the particle density of a system exceeds a critical, temperature-dependent threshold, the particle–ZPF interaction comes to the fore and induces a spontaneous reorganization of the system in the course of which the individual behavior of the particles changes into a collective system behavior. The particle–ZPF interaction is governed by resonance frequencies arising from the excitation spectra of the involved particle species (Preparata, 1995; Del Giudice et al., 2005). Notably, due to its lowered energy per particle compared to the disordered state, the coherent system state is a stable, energetically favored configuration, giving rise to the release of the abovementioned photon pulse (Dicke, 1954; Preparata, 1995).

As far as cortical phase transitions and attractor formations are concerned, it can be assumed that they are triggered by modulating the density of a particular set of molecules, the excitation spectra of which ultimately determine the set of phase-locked ZPF modes constituting the attractor-specific ZPF information states. The objective of further research activities must be to identify the set of these molecules and to understand their functional interaction. Interfacial water in close proximity to hydrophilic surfaces certainly plays an important role in this molecular interplay as it is effectively shielded against thermal interference and therefore forms extensive coherence regions (Del Giudice et al., 2010, 2013), thus simulating a low-temperature environment and preventing the decoherence of coherent system states.

Even though the theoretical foundations still need to be refined, these initial insights can be used to derive testable predictions and guidelines for the experimental approach. In particular, the recurring phase transitions underlying conscious memory processes should reveal themselves through characteristic photon emissions. In the case of memory formation and conscious perception, such photon emissions are predicted to follow the theta rhythm, while memory retrieval and self-referential conscious processes should exhibit photon pulses correlated with the alpha cycle (Keppler, 2018b). In order to detect these phenomena, also referred to as *ultraweak photon emission* or *biophoton emission*, “highly sensitive measuring instruments are required that allow non-invasive and non-destructive recording,” such as photomultipliers, spectral analyzers, and photon counting devices (Van Wijk and Van Wijk, 2005). The experimental background of these detection methods is well developed (Popp et al., 1994; Cohen and Popp, 1997; Popp, 2003). Experiments of this kind show that the biophoton emission depends on the state of health as well as the state of consciousness of a subject (Van Wijk and Van Wijk, 2005) and changes, for example, during the application of meditation techniques (Van Wijk et al., 2005). There are also first indications that the biophoton emission intensity is correlated with the theta cycle (Kobayashi et al., 1999), which is in line with one of the predictions given above. However, it must be clearly pointed out that in order to provide a solid confirmation of the formulated hypotheses, the expected intensity and spectral characteristics of the emitted photons must first be determined more precisely on the basis of the theoretical considerations presented here. A more detailed analysis of the emission characteristics will allow conclusions to be drawn on the attractor-specific modifications of the ZPF, eventually leading to the systematic calibration of ZPF information states on the basis of first-person accounts (Keppler, 2016). This will open up completely new possibilities for the exploration of memory traces and phenomenal experiences.

## Author Contributions

The author confirms being the sole contributor of this work and has approved it for publication.

## Conflict of Interest

The author declares that the research was conducted in the absence of any commercial or financial relationships that could be construed as a potential conflict of interest.
